# Transporter genes expressed by coastal bacterioplankton in response to dissolved organic carbon

**DOI:** 10.1111/j.1462-2920.2009.02102.x

**Published:** 2010-03

**Authors:** Rachel S Poretsky, Shulei Sun, Xiaozhen Mou, Mary Ann Moran

**Affiliations:** Department of Marine Sciences, University of GeorgiaAthens, GA 30602-3636, USA

## Abstract

Coastal ocean bacterioplankton control the flow of dissolved organic carbon (DOC) from terrestrial and oceanic sources into the marine food web, and regulate the release of inorganic carbon to atmospheric and offshore reservoirs. While the fate of the chemically complex coastal DOC reservoir has long been recognized as a critical feature of the global carbon budget, it has been problematic to identify both the compounds that serve as major conduits for carbon flux and the roles of individual bacterioplankton taxa in mediating that flux. Here we analyse random libraries of expressed genes from a coastal bacterial community to identify sequences representing DOC-transporting proteins. Predicted substrates of expressed transporter genes indicated that carboxylic acids, compatible solutes, polyamines and lipids may be key components of the biologically labile DOC pool in coastal waters, in addition to canonical bacterial substrates such as amino acids, oligopeptides and carbohydrates. Half of the expressed DOC transporter sequences in this coastal ocean appeared to originate from just eight taxa: *Roseobacter*, SAR11, *Flavobacteriales* and five orders of γ-*Proteobacteria*. While all major taxa expressed transporter genes for some DOC components (e.g. amino acids), there were indications of specialization within the bacterioplankton community for others (e.g. carbohydrates, carboxylic acids and polyamines). Experimental manipulations of the natural DOC pool that increased the concentration of phytoplankton- or vascular plant-derived compounds invoked a readily measured response in bacterial transporter gene expression. This highly resolved view of the potential for carbon flux into heterotrophic bacterioplankton cells identifies possible bioreactive components of the coastal DOC pool and highlights differing ecological roles in carbon turnover for the resident bacterial taxa.

## Introduction

Coastal ocean ecosystems process dissolved organic carbon (DOC) from both terrestrial and marine sources, making these complex and productive systems critical to our understanding of the global carbon cycle (Hedges *et al*., 1997). Bacterial processing of coastal DOC remains a significant conceptual and analytical challenge, however. Thousands of compounds make up the DOC pool, each with different biological turnover rates (Cherrier and Bauer, 2004) and many of which no longer resemble the parent biomolecules from which they were formed (Ogawa *et al*., 2001). This complex DOC pool is processed by a diverse community of heterotrophic bacterioplankton composed of hundreds of different taxa (Giovannoni and Stingl, 2005) with varying ecological strategies for the uptake and metabolism of organic carbon (Cottrell and Kirchman, 2000; Mou *et al*., 2008).

Several methodologies can provide insights into biological turnover of DOC in marine waters. Approaches that track changes in substrate concentrations over time (Raymond and Bauer, 2000), or use radiotracers to estimate turnover rates (i.e. the fraction of a compound transformed per unit time) (Wright, 1978; Zubkov *et al*., 2008) can measure fluxes of individual components of DOC into bacterioplankton cells. These studies show important roles for amino acids and monosaccharides (usually glucose) in bacterially mediated DOC turnover; together, these two compound classes can account for ∼40% of total carbon assimilation by bacterial cells (Kirchman, 2003). Approaches measuring DOC uptake at the single-cell level (e.g. Ouverney and Fuhrman, 1999; Cottrell and Kirchman, 2000; Mou *et al*., 2007) show that most marine bacterioplankton are capable of transporting amino acids and glucose (Malmstrom *et al*., 2005) and that phylum- or class-level taxonomic groupings exhibit differential uptake of DOC components, including amino acids, glucose, dimethylsulfoniopropionate, glycine betaine and vanillic acid (Cottrell and Kirchman, 2000; Malmstrom *et al*., 2005; Alonso and Pernthaler, 2006; Mou *et al*., 2008). These methodologies are typically limited, however, to a small number of compounds whose importance as a bacterial substrate must be assumed *a priori*. Thus further work is needed to assemble a comprehensive understanding of the compounds that act as primary conduits for carbon flow in coastal waters, and how these pipelines for DOC processing fluctuate on a spatial and temporal basis.

Here we use a complementary methodology based on functional metagenomics that surveys the DOC pool to identify potentially bioreactive components as well as the taxa that may be responsible for their turnover. We assembled large-scale libraries of mRNA sequences from a coastal bacterioplankton community, identified transcripts involved in the transport of DOC, and analysed the predicted substrates and taxonomic origin of these transporter sequences. Although it cannot furnish quantitative measures of flux, transporter gene expression analysis provides insight into potential uptake of organic compounds by bacterioplankton, including substrates that are in low concentration but high demand (and therefore difficult to assess by direct chemical measures of DOC) and those not presupposed to be important conduits for DOC turnover. Using this functional metagenomics approach, we generate hypotheses about the bioreactive components of south-eastern US coastal DOC and the taxonomic identities and substrate preferences of the bacterial taxa controlling DOC flux.

## Results and discussion

### Transcript libraries

Transcript sequences were obtained in May 2007 from duplicate samples of a bacterioplankton community from south-eastern US coastal seawater (31°22′57.22″N, 81°16′51.19″W) with a DOC concentration of 603 μM. Reverse transcription of mRNA-enriched community RNA followed by amplification and pyrosequencing yielded > 140 000 potential protein-encoding sequences from each sample (Table 1). Using bioinformatic criteria established by *in silico* analyses of known bacterial genes (see *Experimental procedures* for details), ∼40% of the potential protein-encoding sequences had hits to the NCBI RefSeq database and ∼17% could be assigned to a COG (clusters of orthologous groups) functional category (Table 1). The remaining sequences likely represent transcripts from unknown genes or poorly conserved regions of known genes (Poretsky *et al*., 2009), or small RNAs (Shi *et al*., 2009).

**Table 1:** Analysis statistics of bacterioplankton community transcript libraries.

	Coastal 1	Coastal 2	Phyto-DOC	VP-DOC
Number of unique reads	435 219	291 109	405 024	444 832
Average read length	186	186	157	206
Number of rRNAs	230 108	150 603	230 432	225 696
Number of potential proteins	205 108	140 506	174 592	219 136
Number of RefSeq hits^a^	68 327	65 700	107 196	144 514
% RefSeq hits^b^	33	47	61	66
Number of COG hits^c^	40 570	21 133	70 981	88 621
% COG hits^b^	20	15	41	40

a Criteria for annotation were *E*-value ≤ 0.01, amino acid identity ≥ 40% and overlapping length ≥ 23 aa to the corresponding best hit. See *Experimental procedures* for information on how these criteria were determined.

b Expressed as a per cent of potential protein-encoding transcripts.

c Criteria for annotation were *E*-value ≤ 0.1, amino acid identity ≥ 40% and overlapping length ≥ 23 aa to the corresponding best hit.

Coastal 1 and 2 = unmanipulated coastal DOC; Phyto-DOC = amended with DOC derived from four strains of coastal phytoplankton; VP-DOC = amended with DOC derived from senescent *Spartina alterniflora*, the dominant vascular plant in adjacent coastal marshes.

### DOC-related transporters

**Fig. 1:**
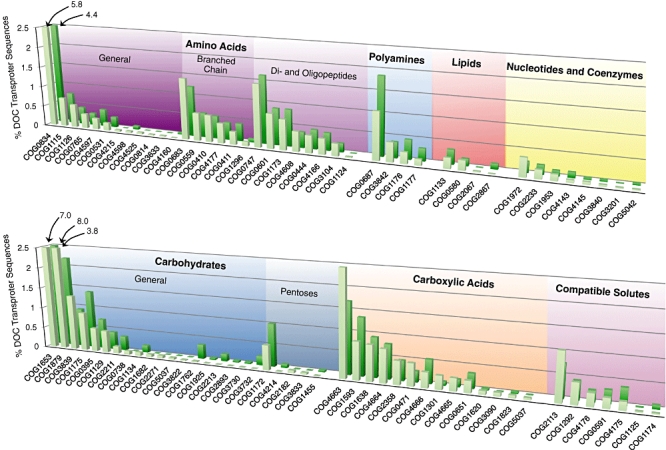
DOC-related transporter COGs expressed in a south-eastern US coastal bacterioplankton community. Two independent replicate samples (Coastal 1 = light green; Coastal 2 = dark green) were collected at night on 6 May 2007, processed for mRNA extraction and amplification, and sequenced using the 454 FLX system. COGs are shown if either of the replicate coastal samples had at least one sequence. COG functional descriptions are as follows: ***Amino Acids, general:*** COG0834, ABC-type amino acid transport/signal transduction systems, periplasmic component/domain; COG1115, Na+/alanine symporter; COG1126, ABC-type polar amino acid transport system, ATPase component; COG0765, ABC-type amino acid transport system, permease component; COG4597, ABC-type amino acid transport system, permease component; COG0531, Amino acid transporters; COG4215, ABC-type arginine transport system, permease component; COG4598, ABC-type histidine transport system, ATPase component; COG4525, ABC-type taurine transport system, ATPase component; COG0814, Amino acid permeases; COG3633, Na+/serine symporter; COG4160, ABC-type arginine/histidine transport system, permease component; ***Amino Acids, branched chain:*** COG0683, ABC-type branched-chain amino acid transport systems, periplasmic component; COG0559, Branched-chain amino acid ABC-type transport system, permease components; COG0410, ABC-type branched-chain amino acid transport systems, ATPase component; COG4177, ABC-type branched-chain amino acid transport system, permease component; COG0411, ABC-type branched-chain amino acid transport systems, ATPase component; COG1296, Predicted branched-chain amino acid permease (azaleucine resistance); ***Amino Acids, di- and oligo-peptides:*** COG0747, ABC-type dipeptide transport system, periplasmic component; COG0601, ABC-type dipeptide/oligopeptide/nickel transport systems, permease components; COG1173, ABC-type dipeptide/oligopeptide/nickel transport systems, permease components; COG4608, ABC-type oligopeptide transport system, ATPase component; COG0444, ABC-type dipeptide/oligopeptide/nickel transport system, ATPase component; COG4166, ABC-type oligopeptide transport system, periplasmic component; COG3104, Dipeptide/tripeptide permease; COG1124, ABC-type dipeptide/oligopeptide/nickel transport system, ATPase component; ***Polyamines:*** COG0687, Spermidine/putrescine-binding periplasmic protein; COG3842, ABC-type spermidine/putrescine transport systems, ATPase components; COG1176, ABC-type spermidine/putrescine transport system, permease component I; COG1177, ABC-type spermidine/putrescine transport system, permease component II; ***Lipids:*** COG1133, ABC-type long-chain fatty acid transport system, fused permease and ATPase components; COG0580, Glycerol uptake facilitator and related permeases (Major Intrinsic Protein Family); COG2067, Long-chain fatty acid transport protein; COG2867, Oligoketide cyclase/lipid transport protein; ***Nucleotides and Coenzymes:*** COG1972, Nucleoside permease; COG2233, Xanthine/uracil permeases; COG1953, Cytosine/uracil/thiamine/allantoin permeases; COG4143, ABC-type thiamine transport system, periplasmic component; COG4145, Na+/panthothenate symporter; COG3840, ABC-type thiamine transport system, ATPase component; COG3201, Nicotinamide mononucleotide transporter; COG5042, Purine nucleoside permease; ***Carbohydrates, general:*** COG1653, ABC-type sugar transport system, periplasmic component; COG1879, ABC-type sugar transport system, periplasmic component; COG3839, ABC-type sugar transport systems, ATPase components; COG1175, ABC-type sugar transport systems, permease components; COG0395, ABC-type sugar transport system, permease component; COG1129, ABC-type sugar transport system, ATPase component; COG2211, Na+/melibiose symporter and related transporters; COG0738, Fucose permease; COG1134, ABC-type polysaccharide/polyol phosphate transport system, ATPase component; COG1682, ABC-type polysaccharide/polyol phosphate export systems, permease component; COG2271, Sugar phosphate permease; COG5037, Gluconate transport-inducing protein; COG3822, ABC-type sugar transport system, auxiliary component; COG1762, Phosphotransferase system mannitol/fructose-specific IIA domain (Ntr-type); COG1925, Phosphotransferase system, HPr-related proteins; COG2213, Phosphotransferase system, mannitol-specific IIBC component; COG2893, Phosphotransferase system, mannose/fructose-specific component IIA; COG3730, Phosphotransferase system sorbitol-specific component IIC; COG3732, Phosphotransferase system sorbitol-specific component IIBC; ***Carbohydrates, pentoses:*** COG1172, Ribose/xylose/arabinose/galactoside ABC-type transport systems, permease components; COG4214, ABC-type xylose transport system, permease component; COG2182, Maltose-binding periplasmic proteins/domains; COG3833, ABC-type maltose transport systems, permease component; COG1455, Phosphotransferase system cellobiose-specific component IIC; ***Carboxylic Acids:*** COG4663, TRAP-type mannitol/chloroaromatic compound transport system, periplasmic component; COG1593, TRAP-type C4-dicarboxylate transport system, large permease component; COG1638, TRAP-type C4-dicarboxylate transport system, periplasmic component; COG4664, TRAP-type mannitol/chloroaromatic compound transport system, large permease component; COG2358, TRAP-type uncharacterized transport system, periplasmic component; COG0471, Di- and tricarboxylate transporters; COG4666, TRAP-type uncharacterized transport system, fused permease components; COG1301, Na+/H+-dicarboxylate symporters; COG4665, TRAP-type mannitol/chloroaromatic compound transport system, small permease component; COG0651, Formate hydrogenlyase subunit 3/Multisubunit Na+/H+ antiporter, MnhD subunit; COG1620, l-lactate permease; COG3090, TRAP-type C4-dicarboxylate transport system, small permease component; COG1823, Predicted Na+/dicarboxylate symporter; COG5037, Gluconate transport-inducing protein; ***Compatible Solutes:*** COG2113, ABC-type proline/glycine betaine transport systems, periplasmic components; COG1292, Choline-glycine betaine transporter; COG4176, ABC-type proline/glycine betaine transport system, permease component; COG0591, Na+/proline symporter; COG4175, ABC-type proline/glycine betaine transport system, ATPase component; COG1125, ABC-type proline/glycine betaine transport systems, ATPase components; COG1174, ABC-type proline/glycine betaine transport systems, permease component.

Transporter-related sequences accounted for 13% of COG annotations, approximately half of which were assigned to categories relevant to DOC consumption (Table S1). Thus about 1 in 20 COG-annotated mRNAs were involved in the uptake of organic molecules by bacterioplankton cells. Predicted substrates for these DOC-related transporters were amino acids (25%; including branched chain and polar amino acids), oligopeptides (11%), carbohydrates (34%), carboxylic acids (16%), compatible solutes such as glycine betaine and proline (5%), polyamines (2%) and lipids (1%) (Fig. 1). Most DOC-related transporter genes expressed by the bacterioplankton community were for components of ATP-binding cassette (ABC) transporters, followed by tripartite ATP-independent periplasmic (TRAP) transporters, and Na+ symporters (Fig. 1); transcripts for phosphotransferase system (PTS) transporters were not common. The independently collected and processed duplicate samples produced highly similar transcript profiles (Fig. 1).

The taxonomic origin of expressed DOC transporter sequences was conservatively inferred from a blastx analysis against the NCBI RefSeq database (Huson *et al*., 2007). Forty-seven per cent of all DOC transporter sequences were assigned to just eight order-level marine bacterial taxa: *Rhodobacterales* (30%; α-*Proteobacteria*, *Primarily Roseobacter*), *Rickettsiales* (10%; α-*Proteobacteria*, primarily SAR11), *Flavobacteriales* (1%), and five orders of γ-*Proteobacteria* (6%; sum of *Alteromonadales*, *Oceanospirallales*, *Pseudomonadales*, *Vibrionales*, and an uncharacterized taxon related to sulfur-oxidizing symbionts). The remaining DOC transporter sequences (53%) were either assigned to one of 12 orders that each accounted for < 1% of sequences or could not be confidently assigned at the order level. The inferred taxonomy of expressed DOC transporters is consistent with a companion PCR-amplified 16S rRNA clone library in which 51% of the sequences were affiliated with these same eight groups: SAR11 (19%), *Roseobacter* (12%), *Flavobacteriales* (11%) and five γ-*Proteobacteria* orders (9%) (Fig. S1). About 20% of the 16S rRNA sequences were classified into taxa without a reference genome sequence (e.g. SAR86, SAR116 and SAR432). In our bioinformatic pipeline, transcripts from these groups would likely be classified only to phylum level (Poretsky *et al*., 2009) and thus are without order-level taxonomic assignments.

**Fig. 2:**
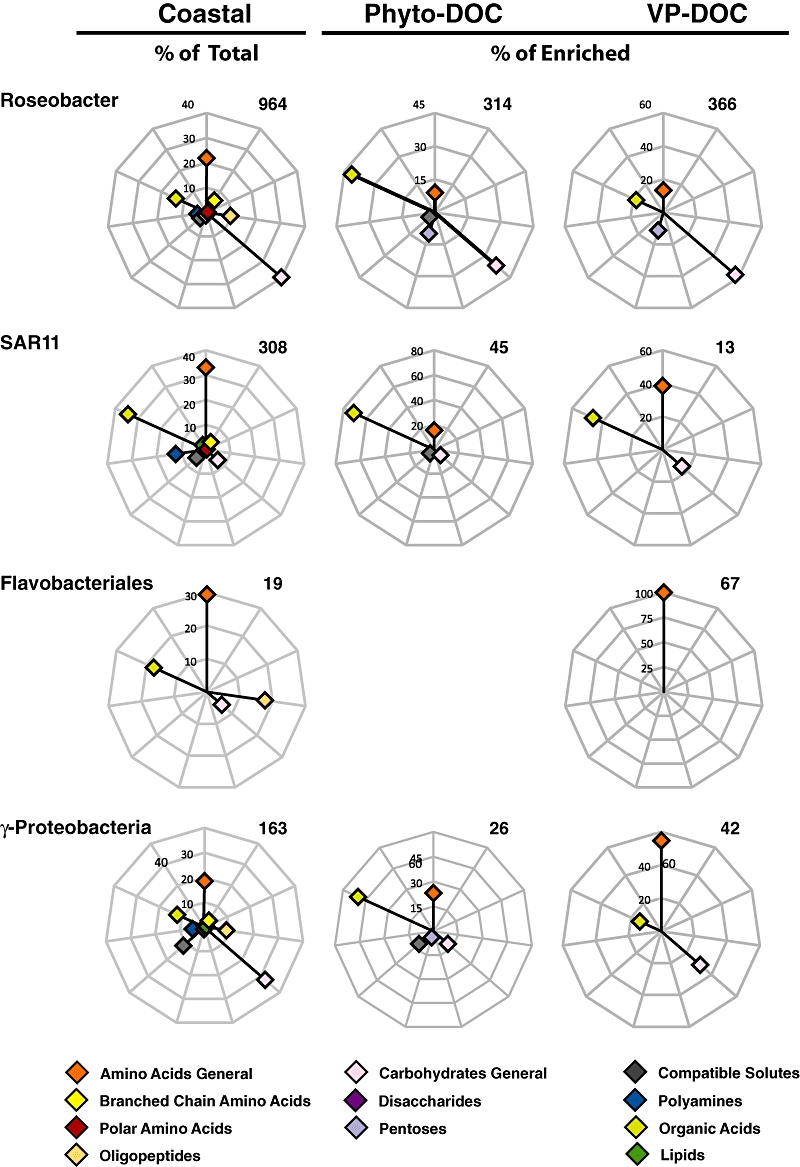
Taxon-specific expression patterns for DOC-related transporter genes. Left column: all DOC-related sequences in unmanipulated coastal seawater (pooled Coastal 1 and Coastal 2 samples). Middle and right columns: significantly enriched DOC-related sequences in coastal seawater amended with phytoplankton-derived DOC (middle) and vascular plant-derived DOC (right). Each point of the radar plot represents the per cent of transporter sequences assigned to a given taxon that was annotated for uptake of the indicated compound class. The number of DOC-related transcript sequences included in the analysis is indicated in the upper right of each plot.

Expression profiles indicated that *Roseobacter* (the taxon with the most DOC-related transporter sequences) and γ-*Proteobacteria* expressed transporter genes for all major classes of organic compounds and may therefore be DOC generalists (Fig. 2). This is in agreement with a previous study in this same coastal ocean showing a diversity of carbon-processing capabilities in bacterial assemblages selected for their ability to metabolize only a single component of the DOC pool (Mou *et al*., 2008). Yet while the absence of strict specialization agrees with the idea that heterogeneity in the supply rate and composition of DOC to coastal oceans may favour generalist bacteria (Mou *et al*., 2008), some order-level taxonomic groupings appeared to have substrate preferences. The *Roseobacter* and γ-*Proteobacteria* groups together accounted for 44% of all carbohydrate-related transporter sequences (and 83% of those that could be taxonomically assigned) and may have dominated carbohydrate turnover in this system at the time of sampling. SAR11 members expressed numerous transporters for carboxylic acids (34% of SAR11-like transporter sequences) and amino acids (37%), but very few for carbohydrates (6%) (Fig. 2). *Flavobacteriales* transporters for the uptake of inorganic compounds (sodium, sulfate, metals) were abundant (Table S1), but few *Flavobacteriales*-like sequences for organic monomer uptake could be recognized in our annotation pipeline. All eight major bacterioplankton taxa expressed genes for amino acid uptake (Fig. 2).

### Concentration versus flux

The abundance of bacterioplankton transcripts that mediate uptake of amino acids (36% of DOC-related transporter sequences) and sugars (34%) (Table 2) contrasts with typical measured concentrations of hydrolysable amino acids (3%) and carbohydrates (7%) in marine DOC (Benner, 2002). Carboxylic acids, polyamines and compatible solutes were likewise considerably better represented in the bacterioplankton transcriptome (17%, 2% and 5% of DOC-related transporters) than in the marine DOC reservoir (5%, < 0.1%, < 1%) (Höfle, 1984; Thurman, 1985; Lee and Jorgensen, 1995; Kiene *et al*., 2000; Benner, 2002). These discrepancies are expected, since rapid consumption of freshly produced DOC by heterotrophic bacterioplankton (Kirchman *et al*., 1991) results in a DOC reservoir that is biased towards biologically recalcitrant constituents (Benner, 2002; Mopper *et al*., 2007). Transporter expression analysis is therefore likely to be a better proxy for the flux of DOC components into bacterioplankton cells than for the chemical composition of seawater DOC (assuming mRNAs generally lead to transporter synthesis and transporter abundance is roughly proportional to uptake). Indeed, transcript abundance correlates considerably better with the chemical composition of the biologically labile DOC pool (e.g. > 20% for amino acids, 10–30% for glucose; Kirchman, 2003) than with the composition of the total DOC pool.

**Table 2:** Bacterioplankton transcripts annotated with functions relevant to DOC uptake.

Organic compound	Coastal 1 % of total	Coastal 2 % of total	Phyto-DOC % of enriched	VP-DOC % of enriched
Amino acids, total	37.6	34.7	12.5	29.6
Amino acids, general	17.6	15.1	12.5	29.6
Amino acids, branched chain	8.3	7.5	0	0
Amino acids, polar	1.3	1.0	0	0
Oligopeptides/dipeptides	10.4	11.1	0	0
Carbohydrates, total	34.1	34.6	24.8	52.5
Carbohydrates, general	32.7	32.1	15.5	45.3
Carbohydrates, disaccharides	0.1	0	0	0
Carbohydrates, pentoses	1.3	2.5	9.3	7.2
Compatible solutes	5.2	5.0	4.4	0
Polyamines	2.0	2.6	4.4	0
Carboxylic acids	17.8	15.2	53.9	17.8
Lipids	1.1	1.2	0	0
Other^a^	2.3	6.7	0	0

a Nucleic acids, vitamins and organic cofactors.

Units are per cent of total DOC-related transporter sequences (Coastal 1 and Coastal 2) or per cent of significantly enriched DOC-related transporter sequences relative to coastal samples (Phyto-DOC and VP-DOC). Phyto-DOC = amended with DOC derived from four strains of coastal phytoplankton; VP-DOC = amended with DOC derived from senescent *Spartina alterniflora*, the dominant vascular plant in adjacent coastal marshes.

Significant mismatches are nonetheless expected between measures of transporter gene expression and the relative fluxes of organic molecules from the DOC pool into heterotrophic bacterioplankton cells. Constitutively expressed transporter genes yield transcripts regardless of substrate availability (although many bacterioplankton transporters were inducible by changes in the DOC pool; see below). Effects of post-transcriptional regulatory mechanisms, transporter affinity, transporter half-life (typically several hours; Cho *et al*., 1981; Solana *et al*., 2001) and substrate concentration similarly complicate efforts to infer transport from transcription. For high-molecular-weight polysaccharides and proteins (Amon and Benner, 1996), bacterial consumption will be evident only indirectly, through transporter expression for constituent monomers or oligomers following extracellular cleavage. Finally, inference of bioreactive DOC from metatranscriptomic data is constrained by the accuracy of bacterial transporter annotation. Sequences assigned to ‘general’ or ‘hypothesized’ transporter categories are uninformative (17% of transporter sequences), and some of the more specific annotations may be misleading for protein families that have diverse functions. If the potential protein-encoding sequences that could not be annotated based on similarity to known genes (∼50%; Table 1) contain DOC-related transporters in the same proportion as the annotated sequences, only half the relevant transporter sequences were detected here.

### DOC-induced transcriptional responses

**Fig. 3:**
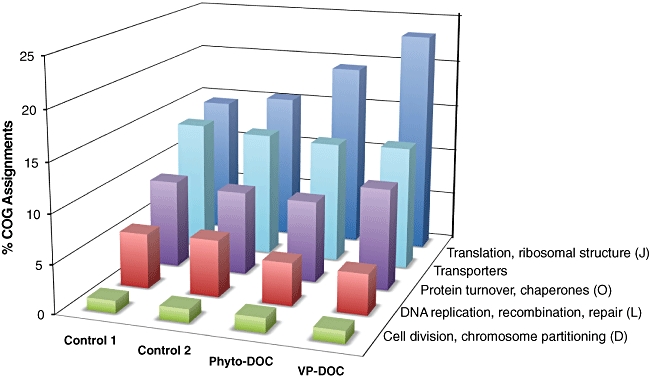
Transcripts assigned to selected major functional categories as a per cent of total COG assignments (COG J only; translation and ribosomal structure) or as a per cent of COG assignments after removal of COG J sequences (COG O, protein turnover and chaperones; COG L, DNA replication, recombination, and repair; COG D, cell division and chromosome partitioning). The ‘transporter’ category sums transporter-related sequences across major functional categories.

Compositionally different bioreactive DOC should induce different transcriptional responses by a bacterioplankton community. If so, transporter gene expression analysis offers a sensitive bioassay for changes in sources and composition of biologically labile DOC over highly resolved temporal scales (i.e. minutes to hours) useful for addressing dynamics in bacterially mediated carbon flux through the coastal DOC pool. We tested this using coastal seawater samples collected in parallel with the unmanipulated samples described above but amended for 1 h with model coastal DOC derived from phytoplankton or vascular plants prior to RNA extraction. The addition of ∼250 μM model DOC to a ∼600 μM DOC background increased the relative abundance of transcripts mediating protein synthesis, as evidenced by upregulation of genes required for synthesis of ribosomes, t-RNAs, and associated initiation, elongation and termination factors (COG J; Fig. 3). This functional category, which typically represents the first macromolecular pool to respond to a shift-up in bacterial activity (Ingraham *et al*., 1983; Chin-Leo and Kirchman, 1990), accounted for 14% and 15% of COG assignments for unmanipulated coastal seawater, but 23% and 19% following DOC amendments (Fig. 3). COG assignments for DNA replication and cell division did not increase (COGs L and D; Fig. 3). These data suggest that a transcriptional response to the DOC additions took place by the end of the 1 h incubation while increases in bacterial cell division rates, an indication of possible changes in the composition of the community gene pool that might complicate data interpretation, had not.

Relative abundance of transporter-related sequences expressed in experimentally modified DOC compared with unmanipulated coastal DOC was assessed using an M–A (magnitude–amplitude) plot (Yang *et al*., 2002). This plot was developed for microarray analysis, and is used here to evaluate the abundance ratio for a COG between the two samples (log abundance ratio; M) against the overall abundance of that COG in both samples (mean log abundance; A). A statistical resampling technique was used to distinguish the transporter COGs for which there were significant differences in relative abundance following amendment (*P*< 0.05) (Rodriguez-Brito *et al*., 2006). For the two replicate unmanipulated coastal samples (independently collected, processed and sequenced), the pair-wise analysis found that only 2% of populated transporter COGs (5 out of 234) were significantly different (Fig. S2).

**Fig. 4:**
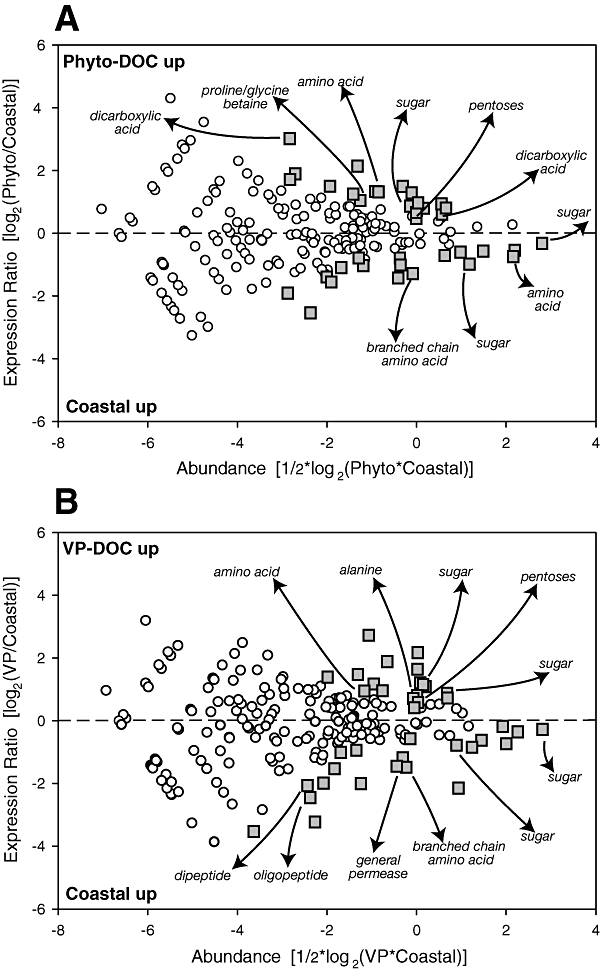
M–A plots of pair-wise comparisons of transcript relative abundance in transporter-related COGs for the combined coastal sample versus phytoplankton-derived DOC (A) and vascular plant-derived DOC (B). M = log_2_(transcript_E_/transcript_C_) and A = 0alf; log_2_(transcript_E_ + transcript_C_), where transcript_E_ and transcript_C_ are the per cent of transcripts assigned to a COG in experimentally manipulated and coastal treatments respectively. Grey squares indicate COGs that are significantly different between samples. See Fig. 2B for the pair-wise comparison between the two experimentally manipulated DOC pools.

In contrast, 15% of the transporter COGs were statistically different in pair-wise comparisons between the pooled coastal samples versus the phytoplankton-derived DOC amendment (39 out of 253 transporter COGs; Fig. 4A) and 16% in pair-wise comparisons between the pooled coastal samples versus the vascular plant-derived DOC amendment (39 out of 250; Fig. 4B). Since coastal samples and experimentally manipulated treatments share a common DOC background, significantly enriched transporters in the experimental treatments signal a substrate-induced response to labile compounds introduced with the DOC amendments. Transcript annotations suggested that bacterioplankton exposed to fresh phytoplankton-derived labile DOC (defined here as the portion consumed within a 1 h period) expressed considerably more genes for the transport of carboxylic acids and fewer for the transport of some classes of carbohydrates relative to coastal DOC (Table 2, Fig. 4A). Cells exposed to fresh vascular plant-derived labile DOC expressed more genes for the transport of carbohydrates (possibly as maltose, fructose, pentoses and mannitol) and fewer for amino acids, peptides and compatible solutes (proline, glycine betaine) (Table 2, Fig. 4B). Time-series analyses in future studies could provide additional information on the order in which components of the model DOC pools invoke the cells' transporter expression response. These bioreactive compound profiles should be informative about typical inputs of fresh DOC at this coastal site, which is largely derived from phytoplankton (Pomeroy *et al*., 1981), benthic algae (Porubsky *et al*., 2008) and *Spartina alterniflora*-dominated marshes (Pakulski, 1986; Moran and Hodson, 1990). Further, they agree with previous research showing that organic acids (Hellebust, 1965), sugars and amino acids (Meon and Kirchman, 2001; Cherrier and Bauer, 2004) are rapidly consumed from plankton-derived DOC, while sugars (particularly glucose and xylose) (Opsahl and Benner, 1997) are the major bioreactive component of *S. alterniflora* leachate.

To determine which bacterioplankton taxa were most responsive to changes in the DOC pool, we inferred the taxonomic origin of sequences assigned to significantly enriched DOC-related transporter COGs (see above). *Roseobacter*-like sequences accounted for most of the significantly enriched transporter sequences to which an order-level taxonomy could be assigned (80% in phytoplankton exudate and 72% in vascular plant leachate). SAR11 sequences were relatively more important among transporter sequences from fresh phytoplankton-derived organic matter than from vascular plant leachate (12% versus 2% of enriched DOC-related transporter sequences), while *Flavobacteriales* showed the opposite pattern (0% versus 14%). Since substrate transport is the first step of many downstream cellular processes, this taxonomic pattern of activity should be mirrored in other (i.e. non-transport-related) functional gene categories; this was found to be the case (Figs S3 and S4). Transporter expression profiles for individual bacterioplankton taxa in experimental treatments showed specialization on DOC compound classes that was similar to that in unmanipulated coastal DOC (Fig. 2).

### Concluding remarks

Determining which components of the marine DOC pool serve as conduits for bacterially mediated carbon flux, and how they vary over time and space, has long been a central challenge for microbial ecology (Kirchman *et al*., 1991; Amon and Benner, 1996; Raymond and Bauer, 2000). The analysis of coastal bacterioplankton transporters provides a new perspective on this important question by identifying labile compounds being detected or anticipated by bacteria. These include many low-concentration/high-flux compounds that could challenge the detection limits of chemical analyses in seawater and would be difficult to measure simultaneously (Fig. 1), as well as compounds that are not typically evaluated for their role in DOC turnover (e.g. carboxylic acids, nucleic acid constituents and polyamines) (Table 2). For example, one out of six COG-annotated DOC transporter sequences encoded proteins for carboxylic acid uptake (Table 2), indicating a major role in carbon flux for a poorly studied compound class with both planktonic (Hellebust, 1965) and photochemical (Moran and Zepp, 1997; Bertilsson and Tranvik, 1998) sources. Improved understanding of the specific substrates targeted by bacterial transporter proteins will allow more information to be extracted from community transcript analysis. Further, this approach can work synergistically with developing methods for high-resolution chemical characterization of DOC (including mass spectrometry and nuclear magnetic resonance spectroscopy; Mopper *et al*., 2007; Sleighter and Hatcher, 2009) to simultaneously identify and assess the bioavailability of thousands of distinct compounds.

**Fig. 5:**
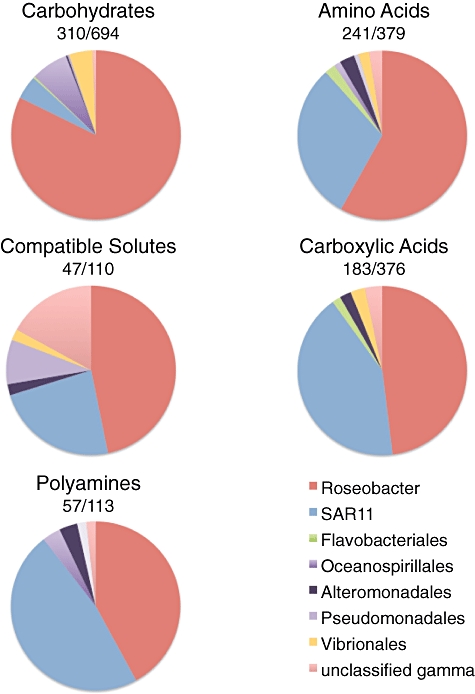
Taxonomic assignment of transporters by DOC compound class. Carbohydrate = transcripts assigned to the general carbohydrate category in Fig. 1; Amino acids = transcripts assigned to the general amino acid category in Fig. 1. Notations above each pie diagram = the number of transcripts included in the pie diagram (i.e. those assigned to one of the eight major taxa)/total transcripts (including minor groups and those not taxonomically assigned at the order level).

To the extent that transporter mRNAs can be considered proxies for DOC flux across bacterioplankton cell membranes, our analysis suggests that two taxa dominated bacterial uptake of DOC in this system at the time it was sampled. Members of the *Roseobacter* group produced 30% of DOC-related transporter sequences and may have been responsible for the bulk of carbohydrate transport, while SAR11 members produced about 10% and showed a preference for carboxylic acids, amino acids and polyamines (Fig. 5); both appeared active in amino acid turnover. Since about half the DOC-related transporter sequences were not assigned to an order-level taxon, there may be other important groups involved or there may be additional sequences from these taxa that did not meet the annotation cut-offs set here. Future sampling efforts that are temporally resolved will provide much-needed insights into the dynamics of bioreactive DOC in this coastal ecosystem, exploring changes in transporter gene expression patterns over daily, monthly and annual cycles. Despite constraints imposed by annotation accuracy and sequencing depth, this analysis of community gene expression provides a novel perspective on rapidly cycling components of coastal DOC, detailed information on the identity and ecological strategies of the bacterial taxa that drive it, and biological detail at a level crucial for future predictive carbon cycle models.

## Experimental procedures

### Sample collection

Coastal water samples were collected in May 2007 at night and high tide from Sapelo Island, GA. Surface water (10–15 l) was passed in succession through a 5.0-μm-pore-size polypropylene cartridge filter (USFilter, Warrendale, PA), a 3.0-μm-pore-size Poretics polycarbonate membrane (Osmonics, Livermore, CA) and a 0.22-μm-pore-size Poretics polycarbonate membrane. Total filtration time was ∼30 min. Two replicate samples were obtained in succession.

### RNA extraction and processing

The 0.22-μm-pore-size filters were vortexed for 10 min in the presence of RNase-free beads (PowerSoil Total RNA Isolation Kit, MoBio, Carlsbad, CA) and lysis/binding solution (RNAqueous-Midi kit; Ambion, Austin, TX). Samples were centrifuged (10 000 r.p.m., 10 min), and supernatants mixed with ethanol solution (RNAqueous-Midi kit). The mixture was passed repeatedly through an 18-gauge needle, filtered through a glass-fibre filter unit (RNAqueous-Midi kit), and washed and eluted according to the manufacturer's instructions. On average, 10 μg of RNA was obtained from each filter. RNA was frozen immediately in liquid nitrogen.

RNA was treated with DNase (TURBO DNA-free kit; Ambion). To minimize rRNA, samples were treated with the mRNA-ONLY Prokaryotic mRNA Isolation Kit (Epicentre Biotechnologies, Madison, WI) followed by the MICROBExpress kit (Ambion). Approximately 500 ng of RNA was linearly amplified (MessageAmp II-Bacteria Kit; Ambion), and amplified antisense RNA (aRNA) was converted to double-stranded cDNA with random hexamers (Universal RiboClone cDNA Synthesis System; Promega, Madison, WI) and purified (Wizard DNA Clean-up System; Promega). cDNAs were sequenced at the Joint Genome Institute using the 454 FLX sequencing system. The sequences have been deposited in the NCBI Short Read Archive with the Genome Project ID #33823.

### Small-submit rRNA clone library construction

DNA was extracted from filters (PowerMax Soil Mega Prep DNA Isolation Kit; MoBio) and small-submit rRNA libraries were constructed following the JGI recommended protocol (http://my.jgi.doe.gov/general/index.html). The sequences have been deposited at NCBI under the Accession No. FJ744762–FJ745272.

### cDNA sequence annotation

Ribosomal RNA sequences were eliminated following identification by blastn queries against the GenBank nucleotide database (nt) as described previously (Mou *et al*., 2008). Criteria for annotation of unassembled potential protein-encoding sequences by blastx analysis against the NCBI non-redundant reference sequence database (RefSeq) were established with *in silico* tests of randomly fragmented full-length known genes with high similarity to metagenomic sequences from Sapelo Island coastal water (Poretsky *et al*., 2009). Based on these analyses, the cut-off criteria were set at *E*-value ≤ 0.01, similarity ≥ 40%, and overlapping length ≥ 65 bp to the corresponding best hit. blastx analysis was also carried out against the COG database using similar cut-off criteria, except the *E*-value was set at ≤ 0.1. COG categories annotated with functions involved in the uptake of organic compounds were identified for further analysis of DOC-related transporters. Taxonomic binning of sequences was carried out using MEGAN (Huson *et al*., 2007). Further assignment into higher-resolution marine taxa (e.g. *Roseobacter* and SAR11) was based on the NCBI taxonomy of closest blast hits.

### Statistical analysis

A resampling program for metagenomic data sets (Rodriguez-Brito *et al*., 2006) was used to compare coastal and experimental sequences categorized based on COG assignments, both for all COG categories and for the subset of COGs representing DOC transport-related functions. The significance level (*P*) was set at < 0.05.

### Transcriptional responses to phytoplankton- and vascular plant-derived DOC

Two additional samples, collected in parallel with those described above, were processed 1 h after amendment with model DOC preparations from coastal phytoplankton and vascular plants. Axenic cultures of marine phytoplankton (*Skeletonema costatum*, CCMP1332; *Chaetoceros calcitrans*, CCMP1315; *Alexandrium tamarense*, CCMP1771; and *Synechococcus* strain WH8101) were grown on a 2- to 3-week schedule at 20°C with a 13:11 light : dark regime of approximately 100 μmol photons m^−2^ s^−1^. To obtain concentrated organic matter for experimental manipulations, stationary-phase cells were filtered onto combusted GF/F filters, homogenized, and filtered again to remove cell and filter debris. The chemical composition of intracellular organic compounds extracted from living phytoplankton has been shown previously to be similar to material released extracellularly by healthy cells (Hellebust, 1965). Senescent (standing dead) *S. alterniflora* culms were collected from Sapelo Island, GA, rinsed with sterile deionized water and ground into fragments. Leachate was prepared by incubation in 10 l of sterile artificial seawater in the dark for 5 days followed by removal of cell debris. DOC concentration was measured by high-temperature catalytic oxidation. Additions of phytoplankton- and vascular plant-derived DOC were made to 10 l of coastal water (250 μM final concentration). After 1 h *in situ* incubation, water samples were filtered as described above.

## Supporting information

**Fig. S1.** Taxonomic assignments of small-subunit rRNA genes amplified from DNA extracted from unmanipulated south-eastern US coastal seawater (data from Coastal 1 sample).

**Fig. S2.** M–A plots of pair-wise comparisons of transcript relative abundance in transporter-related COGs for duplicate coastal samples (A) and phytoplankton- versus vascular plant-derived DOC (B). M = log_2_(transcript_E_/transcript_C_) and A = ½ log_2_(transcript_E_ + transcript_C_), where transcript_E_ and transcript_C_ are the per cent of transcripts assigned to a COG in experimentally manipulated and coastal treatments respectively. Grey squares indicate COGs that are significantly different between samples.

**Fig. S3.** Heat map of transcript abundance for COGs significantly enriched in phytoplankton-derived DOC relative to the pooled unmanipulated coastal samples. Red arrows indicate significantly enriched COGs with DOC-related transporter functions.

**Fig. S4.** Heat map of transcript abundance for COGs significantly enriched in vascular plant-derived DOC relative to the pooled unmanipulated coastal samples. Red arrows indicate significantly enriched COGs with DOC-related transporter functions.

**Table S1.** Number of bacterioplankton transcript sequences assigned to transporter-related COGs. The library size for each sample differs (see Table 1).

## References

[b1] Alonso C, Pernthaler J (2006). *Roseobacter* and SAR11 dominate microbial glucose uptake in coastal North Sea waters. Environ Microbiol.

[b2] Amon RMW, Benner R (1996). Bacterial utilization of different size classes of dissolved organic matter. Limnol Oceanogr.

[b3] Benner R, Hansell D, Carlson C (2002). Chemical composition and reactivity. Biogeochemistry of Marine Dissolved Organic Matter.

[b4] Bertilsson S, Tranvik LJ (1998). Photochemically produced carboxylic acids as substrates for freshwater bacterioplankton. Limnol Oceanogr.

[b5] Cherrier J, Bauer JE (2004). Bacterial utilization of transient plankton-derived dissolved organic carbon and nitrogen inputs in surface ocean waters. Aquat Microb Ecol.

[b6] Chin-Leo G, Kirchman DL (1990). Unbalanced growth in natural assemblages of marine bacterioplankton. Mar Ecol Prog Ser.

[b7] Cho B-H, Sauer N, Komor E, Tanner W (1981). Glucose induces two amino acid transport systems in *Chlorella*. Proc Natl Acad Sci USA.

[b8] Cottrell MT, Kirchman DL (2000). Natural assemblages of marine proteobacteria and members of the *Cytophaga–Flavobacter* cluster consuming low- and high-molecular-weight dissolved organic matter. Appl Environ Microbiol.

[b9] Giovannoni SJ, Stingl U (2005). Molecular diversity and ecology of microbial plankton. Nature.

[b10] Hedges JI, Keil RG, Benner R (1997). What happens to terrestrial organic matter in the ocean?. Org Geochem.

[b11] Hellebust JA (1965). Excretion of some organic compounds by marine phytoplankton. Limnol Oceanogr.

[b12] Höfle MG (1984). Degradation of putrescine and cadaverine in seawater cultures by marine bacteria. Appl Environ Microbiol.

[b13] Huson DH, Auch AF, Qi J, Schuster SC (2007). MEGAN analysis of metagenomic data. Genome Res.

[b14] Ingraham JL, Maaløe O, Neidhardt FC (1983). Growth of the Bacterial Cell.

[b15] Kiene RP, Linn LJ, Bruton JA (2000). New and important roles for DMSP in marine microbial communities. J Sea Res.

[b16] Kirchman DL, Findlay SEG, Sinsabaugh RL (2003). The contribution of monomers and other low-molecular weight compounds to the flux of dissolved organic material in aquatic ecosystems. Aquatic Ecosystems: Interactivity of Dissolved Organic Matter.

[b17] Kirchman DL, Suzuki Y, Garside C, Ducklow HW (1991). High turnover rates of dissolved organic carbon during a spring phytoplankton bloom. Nature.

[b18] Lee C, Jorgensen NOG (1995). Seasonal cycling of putrescine and amino-acids in relation to biological production in a stratified coastal salt pond. Biogeochem.

[b19] Malmstrom RR, Cottrell MT, Elifantz H, Kirchman DL (2005). Biomass production and assimilation of dissolved organic matter by SAR11 bacteria in the Northwest Atlantic Ocean. Appl Environ Microbiol.

[b20] Meon B, Kirchman DL (2001). Dynamics and molecular composition of dissolved organic material during experimental phytoplankton blooms. Mar Chem.

[b21] Mopper K, Stubbins A, Ritchie JD, Bialk HM, Hatcher PG (2007). Advanced instrumental approaches for characterization of marine dissolved organic matter: extraction techniques, mass spectrometry, and nuclear magnetic resonance spectroscopy. Chem Rev.

[b22] Moran MA, Hodson RE (1990). Contributions of degrading *Spartina alterniflora* lignocellulose to the dissolved organic carbon pool of a salt marsh. Mar Ecol Prog Ser.

[b23] Moran MA, Zepp RG (1997). Role of photoreactions in the formation of biologically labile compounds from dissolved organic matter. Limnol Oceanogr.

[b24] Mou X, Hodson RE, Moran MA (2007). Bacterioplankton assemblages transforming dissolved organic compounds in coastal seawater. Environ Microbiol.

[b25] Mou X, Sun S, Edwards RA, Hodson RE, Moran MA (2008). Bacterial carbon processing by generalist species in the coastal ocean. Nature.

[b26] Ogawa H, Amagai Y, Koike I, Kaiser K, Benner R (2001). Production of refractory dissolved organic matter by bacteria. Science.

[b27] Opsahl S, Benner R (1997). Distribution and cycling of terrigenous dissolved organic matter in the ocean. Nature.

[b28] Ouverney CC, Fuhrman JA (1999). Combined microautoradiography-16S rRNA probe technique for determination of radioisotope uptake by specific microbial cell types *in situ*. Appl Environ Microbiol.

[b29] Pakulski JD (1986). The release of reducing sugars and dissolved organic carbon from *Spartina alterniflora* Loisel in a Georgia salt marsh. Estuar Coast Shelf S.

[b30] Pomeroy LR, Darley WM, Dunn EL, Gallagher JL, Haines EB, Whitney DM, Pomeroy LR, Wiegert RG (1981). Primary production. The Ecology of a Salt Marsh..

[b31] Poretsky RS, Hewson I, Sun S, Allen AE, Zehr JP, Moran MA (2009). Comparative day/night metatranscriptomic analysis of microbial communities in the North Pacific Subtropical Gyre. Environ Microbiol.

[b32] Porubsky WP, Velasquez LE, Joye SB (2008). Nutrient-replete benthic microalgae as a source of dissolved organic carbon to coastal waters. Estuar Coast.

[b33] Raymond PA, Bauer JE (2000). Bacterial consumption of DOC during transport through a temperate estuary. Aquat Microb Ecol.

[b34] Rodriguez-Brito B, Rohwer F, Edwards R (2006). An application of statistics to comparative metagenomics. BMC Bioinformatics.

[b35] Shi Y, Tyson GW, DeLong EF (2009). Metatranscriptomics reveals unique microbial small RNAs in the ocean's water column. Nature.

[b36] Sleighter RL, Hatcher PG (2009). Molecular characterization of dissolved organic matter (DOM) along a river to ocean transect of the lower Chesapeake Bay by ultrahigh resolution electrospray ionization Fourier transform ion cyclotron resonance mass spectrometry. Mar Chem.

[b37] Solana S, Reglero A, Martínez-Blanco H, Revilla-Nuin B, Bravo IG, Rodríguez-Aparicio LB, Ferrero MA (2001). *N*-Acetylneuraminic acid uptake in *Pasteurella* (*Mannheimia*) *haemolytica* A2 occurs by an inducible and specific transport system. FEBS Lett.

[b38] Thurman EM (1985). Organic Geochemistry of Natural Waters.

[b39] Wright RT (1978). Measurement and significance of specific activity in the heterotrophic bacteria of natural waters. Appl Environ Microbiol.

[b40] Yang YH, Dudoit S, Luu P, Lin DM, Peng V, Ngai J, Speed TP (2002). Normalization of cDNA microarray data: a robust composite model addressing single and multiple slide systematic variation. Nucleic Acids Res.

[b41] Zubkov MV, Tarran GA, Mary I, Fuchs BM (2008). Differential microbial uptake of dissolved amino acids and amino sugars in surface waters of the Atlantic Ocean. J Plankton Res.

