# Supplementary material for: Transporter genes expressed by coastal bacterioplankton in response to dissolved organic carbon
Source: Environ Microbiol. 2010 Mar;12(3):616–27. doi: 10.1111/j.1462-2920.2009.02102.x (PMC2847192; doi:10.1111/j.1462-2920.2009.02102.x)
Supplement: Supplementary file 1 [file emi0012-0616-SD1.pdf]

Transporter Genes Expressed by Coastal Bacterioplankton in Response to Dissolved Organic Carbon

Rachel S. Poretsky, Shulei Sun, Xiaozhen Mou, and Mary Ann Moran

Table S1. Number of bacterioplankton transcript sequences assigned to transporter-related COGs. The total number of sequences differs for each sample (see Table 1).

COG number	COG Category	COG name	Control 1	Control 2	Phyto-DOC	VP-DOC
COG0410	Amino acid transport and metabolism	ABC-type branched-chain amino acid transport systems, ATPase component	31	13	33	43
COG0411		ABC-type branched-chain amino acid transport systems, ATPase component	15	10	38	31
COG0444		ABC-type dipeptide/oligopeptide/nickel transport system, ATPase component	20	11	15	10
COG0531		Amino acid transporters	9	6	7	7
COG0559		Branched-chain amino acid ABC-type transport system, permease components	32	14	54	49
COG0591		Na+/proline symporter	11	7	17	26
COG0601		ABC-type dipeptide/oligopeptide/nickel transport systems, permease components	40	21	50	59
COG0683		ABC-type branched-chain amino acid transport systems, periplasmic component	71	29	45	44
COG0747		ABC-type dipeptide transport system, periplasmic component	72	39	122	106
COG0765		ABC-type amino acid transport system, permease component	17	7	63	68
COG0814		Amino acid permeases	1	0	0	2
COG0834		ABC-type amino acid transport/signal transduction systems, periplasmic component/domain	274	104	263	372
COG1114		Branched-chain amino acid permeases	0	0	1	0
COG1115		Na+/alanine symporter	34	18	80	114
COG1124		ABC-type dipeptide/oligopeptide/nickel transport system, ATPase component	1	0	2	1
COG1125		ABC-type proline/glycine betaine transport systems, ATPase components	1	0	0	1
COG1126		ABC-type polar amino acid transport system, ATPase component	27	10	45	41
COG1173		ABC-type dipeptide/oligopeptide/nickel transport systems, permease components	35	21	50	61
COG1174		ABC-type proline/glycine betaine transport systems, permease component	1	2	2	0
COG1176		ABC-type spermidine/putrescine transport system, permease component I	13	11	25	30
COG1177		ABC-type spermidine/putrescine transport system, permease component II	7	7	25	34
COG1296		Predicted branched-chain amino acid permease (azaleucine resistance)	5	2	6	6

COG2113		ABC-type proline/glycine betaine transport systems, periplasmic components	53	18	57	63
COG3104		Dipeptide/tripeptide permease	14	5	34	31
COG3633		Na+/serine symporter	1	0	3	2
COG3842		ABC-type spermidine/putrescine transport systems, ATPase components	22	9	53	57
COG4160		ABC-type arginine/histidine transport system, permease component	0	1	4	1
COG4166		ABC-type oligopeptide transport system, periplasmic component	18	10	21	33
COG4175		ABC-type proline/glycine betaine transport system, ATPase component	9	9	14	26
COG4176		ABC-type proline/glycine betaine transport system, permease component	14	6	44	20
COG4177		ABC-type branched-chain amino acid transport system, permease component	23	10	42	47
COG4215		ABC-type arginine transport system, permease component	2	0	3	2
COG4597		ABC-type amino acid transport system, permease component	13	10	34	47
COG4598		ABC-type histidine transport system, ATPase component	2	2	6	4
COG4608		ABC-type oligopeptide transport system, ATPase component	23	9	23	7
COG0395	Carbohydrate transport and metabolism	ABC-type sugar transport system, permease component	27	17	89	140
COG0580		Glycerol uptake facilitator and related permeases (Major Intrinsic Protein Family)	11	4	13	18
COG0738		Fucose permease	4	0	11	13
COG1129		ABC-type sugar transport system, ATPase component	25	10	37	60
COG1134		ABC-type polysaccharide/polyol phosphate transport system, ATPase component	4	4	13	8
COG1172		Ribose/xylose/arabinose/galactoside ABC-type transport systems, permease components	28	24	84	97
COG1175		ABC-type sugar transport systems, permease components	44	33	113	187
COG1263		Phosphotransferase system IIC components, glucose/maltose/N-acetylglucosamine-specific	0	0	2	5
COG1264		Phosphotransferase system IIB components	0	0	0	2
COG1299		Phosphotransferase system, fructose-specific IIC component	0	0	0	1
COG1455		Phosphotransferase system cellobiose-specific component IIC	0	1	0	1
COG1593		TRAP-type C4-dicarboxylate transport system, large permease component	42	32	153	100
COG1638		TRAP-type C4-dicarboxylate transport system, periplasmic component	40	22	54	108
COG1653		ABC-type sugar transport system, periplasmic component	334	189	450	584
COG1682		ABC-type polysaccharide/polyol phosphate export systems, permease component	3	0	2	4
COG1879		ABC-type sugar transport system, periplasmic component	182	52	118	159
COG1925		Phosphotransferase system, HPr-related proteins	0	1	9	9
COG2190		Phosphotransferase system IIA components	0	0	2	4
COG2211		Na+/melibiose symporter and related transporters	8	8	5	2

COG2213		Phosphotransferase system, mannitol-specific IIBC component	0	4	3	1
COG2271		Sugar phosphate permease	3	0	0	0
COG2893		Phosphotransferase system, mannitol-specific IIBC component	0	4	3	1
COG3090		TRAP-type C4-dicarboxylate transport system, small permease component	4	0	30	18
COG3730		Phosphotransferase system sorbitol-specific component IIC	0	1	0	1
COG3732		Phosphotransferase system sorbitol-specific component IIBC	0	5	1	3
COG3833		ABC-type maltose transport systems, permease component	2	0	5	8
COG3839		ABC-type sugar transport systems, ATPase components	63	22	131	149
COG4213		ABC-type xylose transport system, periplasmic component	0	0	1	3
COG4214		ABC-type xylose transport system, permease component	0	2	9	7
COG5037		Gluconate transport-inducing protein	2	0	0	1
COG1292	Cell envelope biogenesis, outer membrane	Choline-glycine betaine transporter	23	10	25	39
COG1596		Periplasmic protein involved in polysaccharide export	1	4	6	7
COG1696		Predicted membrane protein involved in D-alanine export	3	5	10	10
COG1732		Periplasmic glycine betaine/choline-binding (lipo)protein of an ABC-type transport system (osmoprotectant binding protein)	0	0	3	1
COG3562		Capsule polysaccharide export protein	0	0	1	1
COG3563		Capsule polysaccharide export protein	0	0	1	2
COG4591		ABC-type transport system, involved in lipoprotein release, permease component	9	5	27	25
COG3225	Cell motility and secretion	ABC-type uncharacterized transport system involved in gliding motility, auxiliary component	2	1	1	3
COG2978	Coenzyme metabolism	Putative p-aminobenzoyl-glutamate transporter	3	0	2	1
COG2998		ABC-type tungstate transport system, permease component	3	2	2	6
COG3201		Nicotinamide mononucleotide transporter	1	0	0	2
COG3840		ABC-type thiamine transport system, ATPase component	2	1	1	2
COG4143		ABC-type thiamine transport system, periplasmic component	3	3	4	9
COG4145		Na+/panthothenate symporter	3	1	0	2
COG4662		ABC-type tungstate transport system, periplasmic component	0	1	2	3
COG0534	Defense Mechanisms	Na+-driven multidrug efflux pump	0	17	29	36
COG0577		ABC-type antimicrobial peptide transport system, permease component	17	12	33	31
COG0842		ABC-type multidrug transport system, permease component	13	9	29	31
COG1131		ABC-type multidrug transport system, ATPase component	47	23	43	59
COG1132		ABC-type multidrug transport system, ATPase and permease components	90	44	87	124
COG1136		ABC-type antimicrobial peptide transport system, ATPase component	59	21	40	63
COG2274		ABC-type bacteriocin/lantibiotic exporters, contain an N-terminal double-glycine peptidase domain	5	4	15	25

COG0651	Energy production and conversion	Formate hydrogenlyase subunit 3/Multisubunit Na+/H+ antiporter, MnhD subunit	13	8	20	21
COG1301		Na+/H+-dicarboxylate symporters	15	2	23	28
COG1347		Na+-transporting NADH:ubiquinone oxidoreductase, subunit NqrD	16	7	60	108
COG1620		L-lactate permease	4	1	0	2
COG1726		Na+-transporting NADH:ubiquinone oxidoreductase, subunit NqrA	19	9	33	57
COG1757		Na+/H+ antiporter	14	9	18	13
COG1805		Na+-transporting NADH:ubiquinone oxidoreductase, subunit NqrB	21	11	98	189
COG1883		Na+-transporting methylmalonyl-CoA/oxaloacetate decarboxylase, beta subunit	4	5	20	25
COG2209		Na+-transporting NADH:ubiquinone oxidoreductase, subunit NqrE	11	3	61	108
COG2851		H+/citrate symporter	0	0	2	1
COG2869		Na+-transporting NADH:ubiquinone oxidoreductase, subunit NqrC	6	4	32	36
COG2871		Na+-transporting NADH:ubiquinone oxidoreductase, subunit NqrF	9	21	105	157
COG3630		Na+-transporting methylmalonyl-CoA/oxaloacetate decarboxylase, gamma subunit	0	0	0	2
COG4987		ABC-type transport system involved in cytochrome bd biosynthesis, fused ATPase and permease components	1	0	0	1
COG4988		ABC-type transport system involved in cytochrome bd biosynthesis, ATPase and permease components	0	0	0	1
COG5295	Extracellular structures	Autotransporter adhesin	5	4	3	2
COG0385	General function prediction only	Predicted Na+-dependent transporter	7	0	5	6
COG0477		Permeases of the major facilitator superfamily	127	44	195	184
COG0488		ATPase components of ABC transporters with duplicated ATPase domains	99	35	104	193
COG0628		Predicted permease	6	13	36	35
COG0679		Predicted permeases	3	1	5	6
COG0697		Permeases of the drug/metabolite transporter (DMT) superfamily	45	25	74	85
COG0701		Predicted permeases	1	0	6	3
COG0730		Predicted permeases	35	17	96	109
COG0733		Na+-dependent transporters of the SNF family	20	7	23	21
COG0795		Predicted permeases	4	1	5	6
COG1033		Predicted exporters of the RND superfamily	4	6	13	7
COG1101		ABC-type uncharacterized transport system, ATPase component	0	0	1	0
COG1123		ATPase components of various ABC-type transport systems, contain duplicated ATPase	17	12	28	27
COG1137		ABC-type (unclassified) transport system, ATPase component	10	14	27	29
COG1277		ABC-type transport system involved in multi-copper enzyme maturation, permease component	2	1	5	6

COG1279	Lysine efflux permease	3	2	4	8
COG1559	Predicted periplasmic solute-binding protein	0	2	10	10
COG1823	Predicted Na+/dicarboxylate symporter	3	1	4	1
COG2215	ABC-type uncharacterized transport system, permease component	3	1	0	1
COG2244	Membrane protein involved in the export of O-antigen and teichoic acid	2	0	3	2
COG2252	Permeases	56	26	67	39
COG2270	Permeases of the major facilitator superfamily	2	6	11	6
COG2358	TRAP-type uncharacterized transport system, periplasmic component	26	11	22	25
COG2391	Predicted transporter component	31	17	105	142
COG2409	Predicted drug exporters of the RND superfamily	1	0	4	0
COG2962	Predicted permeases	4	1	11	12
COG2985	Predicted permease	0	0	1	0
COG3329	Predicted permease	2	0	2	1
COG3822	ABC-type sugar transport system, auxiliary component	1	0	5	5
COG3845	ABC-type uncharacterized transport systems, ATPase components	19	8	21	26
COG4134	ABC-type uncharacterized transport system, periplasmic component	6	1	4	3
COG4135	ABC-type uncharacterized transport system, permease component	3	0	0	0
COG4136	ABC-type uncharacterized transport system, ATPase component	1	0	0	1
COG4137	ABC-type uncharacterized transport system, permease component	0	1	0	1
COG4146	Predicted symporter	9	6	25	27
COG4147	Predicted symporter	186	88	344	319
COG4152	ABC-type uncharacterized transport system, ATPase component	2	2	2	8
COG4158	Predicted ABC-type sugar transport system, permease component	0	0	2	0
COG4172	ABC-type uncharacterized transport system, duplicated ATPase component	24	8	6	19
COG4174	ABC-type uncharacterized transport system, permease component	9	0	11	8
COG4178	ABC-type uncharacterized transport system, permease and ATPase components	0	0	2	0
COG4239	ABC-type uncharacterized transport system, permease component	10	4	14	11
COG4533	ABC-type uncharacterized transport system, periplasmic component	0	0	0	1
COG4586	ABC-type uncharacterized transport system, ATPase component	1	1	1	1
COG4590	ABC-type uncharacterized transport system, permease component	0	0	2	4
COG4603	ABC-type uncharacterized transport system, permease component	8	2	20	23
COG4618	ABC-type protease/lipase transport system, ATPase and permease components	1	1	5	11
COG4666	TRAP-type uncharacterized transport system, fused permease components	17	13	26	14
COG5006	Predicted permease, DMT superfamily	0	1	0	0

COG0004	Inorganic ion transport and metabolism	Ammonia permease	161	70	167	195
COG0025		NhaP-type Na+/H+ and K+/H+ antiporters	2	4	15	20
COG0053		Predicted Co/Zn/Cd cation transporters	7	1	2	11
COG0168		Trk-type K+ transport systems, membrane components	31	16	51	40
COG0226		ABC-type phosphate transport system, periplasmic component	68	21	36	47
COG0306		Phosphate/sulphate permeases	16	6	25	40
COG0310		ABC-type Co2+ transport system, permease component	1	0	2	0
COG0370		Fe2+ transport system protein B	8	3	24	29
COG0387		Ca2+/H+ antiporter	2	0	0	0
COG0428		Predicted divalent heavy-metal cations transporter	5	1	14	12
COG0471		Di- and tricarboxylate transporters	25	6	29	26
COG0474		Cation transport ATPase	35	11	22	6
COG0475		Kef-type K+ transport systems, membrane components	3	6	4	6
COG0530		Ca2+/Na+ antiporter	11	9	35	55
COG0569		K+ transport systems, NAD-binding component	15	13	39	35
COG0573		ABC-type phosphate transport system, permease component	21	8	11	25
COG0581		ABC-type phosphate transport system, permease component	12	11	12	20
COG0598		Mg2+ and Co2+ transporters	2	1	3	2
COG0600		ABC-type nitrate/sulfonate/bicarbonate transport system, permease component	12	4	14	26
COG0609		ABC-type Fe3+-siderophore transport system, permease component	3	0	1	8
COG0614		ABC-type Fe3+-hydroxamate transport system, periplasmic component	3	0	1	1
COG0619		ABC-type cobalt transport system, permease component CbiQ and related transporters	0	0	0	1
COG0651		Formate hydrogenlyase subunit 3/Multisubunit Na+/H+ antiporter, MnhD subunit	13	8	20	21
COG0659		Sulfate permease and related transporters (MFS superfamily)	37	17	90	145
COG0672		High-affinity Fe2+/Pb2+ permease	1	0	0	0
COG0715		ABC-type nitrate/sulfonate/bicarbonate transport systems, periplasmic components	6	5	12	18
COG0725		ABC-type molybdate transport system, periplasmic component	2	0	0	0
COG0798		Arsenite efflux pump ACR3 and related permeases	1	1	10	14
COG0803		ABC-type metal ion transport system, periplasmic component/surface adhesin	2	9	13	14
COG1006		Multisubunit Na+/H+ antiporter, MnhC subunit	1	0	2	4
COG1009		NADH:ubiquinone oxidoreductase subunit 5 (chain L)/Multisubunit Na+/H+ antiporter, MnhA subunit	54	20	147	130
COG1055		Na+/H+ antiporter NhaD and related arsenite permeases	10	6	42	60

COG1108	ABC-type Mn2+/Zn2+ transport systems, permease components	16	9	19	23
COG1116	ABC-type nitrate/sulfonate/bicarbonate transport system, ATPase component	14	10	11	8
COG1117	ABC-type phosphate transport system, ATPase component	11	8	11	16
COG1118	ABC-type sulfate/molybdate transport systems, ATPase component	0	2	1	0
COG1119	ABC-type molybdenum transport system, ATPase component/photorepair protein PhrA	0	0	5	0
COG1120	ABC-type cobalamin/Fe3+-siderophores transport systems, ATPase components	2	2	3	3
COG1121	ABC-type Mn/Zn transport systems, ATPase component	13	7	11	13
COG1122	ABC-type cobalt transport system, ATPase component	4	3	1	2
COG1135	ABC-type metal ion transport system, ATPase component	3	0	2	1
COG1178	ABC-type Fe3+ transport system, permease component	20	17	24	40
COG1226	Kef-type K+ transport systems, predicted NAD-binding component	21	3	14	19
COG1283	Na+/phosphate symporter	8	7	8	3
COG1320	Multisubunit Na+/H+ antiporter, MnhG subunit	0	2	3	0
COG1392	Phosphate transport regulator (distant homolog of PhoU)	0	1	1	2
COG1563	Predicted subunit of the Multisubunit Na+/H+ antiporter	1	0	0	0
COG1629	Outer membrane receptor proteins, mostly Fe transport	259	106	271	268
COG1668	ABC-type Na+ efflux pump, permease component	2	1	0	1
COG1840	ABC-type Fe3+ transport system, periplasmic component	30	12	32	35
COG1863	Multisubunit Na+/H+ antiporter, MnhE subunit	1	1	5	1
COG1914	Mn2+ and Fe2+ transporters of the NRAMP family	5	2	7	6
COG1918	Fe2+ transport system protein A	1	0	1	1
COG1965	Protein implicated in iron transport, frataxin homolog	0	2	0	0
COG2059	Chromate transport protein ChrA	2	3	3	2
COG2072	Predicted flavoprotein involved in K+ transport	28	24	43	18
COG2076	Membrane transporters of cations and cationic drugs	0	0	7	4
COG2111	Multisubunit Na+/H+ antiporter, MnhB subunit	1	0	9	5
COG2116	Formate/nitrite family of transporters	0	0	0	2
COG2212	Multisubunit Na+/H+ antiporter, MnhF subunit	2	1	0	0
COG2217	Cation transport ATPase	23	11	39	32
COG2223	Nitrate/nitrite transporter	0	4	0	0
COG2239	Mg/Co/Ni transporter MgtE (contains CBS domain)	12	8	33	38
COG3004	Na+/H+ antiporter	7	8	33	39
COG3067	Na+/H+ antiporter	15	2	10	6
COG3158	K+ transporter	3	0	0	5

COG3221		ABC-type phosphate/phosphonate transport system, periplasmic component	2	3	1	2
COG3638		ABC-type phosphate/phosphonate transport system, ATPase component	3	0	2	1
COG3639		ABC-type phosphate/phosphonate transport system, permease component	4	1	4	4
COG4107		ABC-type phosphonate transport system, ATPase component	1	0	0	3
COG4148		ABC-type molybdate transport system, ATPase component	2	0	0	3
COG4149		ABC-type molybdate transport system, permease component	0	1	2	5
COG4208		ABC-type sulfate transport system, permease component	0	0	0	1
COG4525		ABC-type taurine transport system, ATPase component	2	0	6	6
COG4531		ABC-type Zn2+ transport system, periplasmic component/surface adhesin	7	4	6	1
COG4535		Putative Mg2+ and Co2+ transporter CorC	2	3	11	16
COG4536		Putative Mg2+ and Co2+ transporter CorB	15	8	11	14
COG4555		ABC-type Na+ transport system, ATPase component	2	1	1	1
COG4594		ABC-type Fe3+-citrate transport system, periplasmic component	0	0	0	1
COG4604		ABC-type enterochelin transport system, ATPase component	0	0	0	1
COG4615		ABC-type siderophore export system, fused ATPase and permease components	1	0	0	1
COG4651		Kef-type K+ transport system, predicted NAD-binding component	0	1	1	6
COG4985		ABC-type phosphate transport system, auxiliary component	0	0	1	0
COG0811	Intracellular trafficking, secretion, and vesicular transport	Biopolymer transport proteins	60	26	123	183
COG0823		Periplasmic component of the Tol biopolymer transport system	16	16	25	44
COG0848		Biopolymer transport protein	18	8	33	28
COG2095		Multiple antibiotic transporter	3	0	10	10
COG3114		Heme exporter protein D	0	0	2	0
COG5101		Importin beta-related nuclear transport receptor	0	1	1	1
COG5122		Transport protein particle (TRAPP) complex subunit	0	0	1	0
COG5347		GTPase-activating protein that regulates ARFs (ADP-ribosylation factors), involved in ARF-mediated vesicular transport	1	0	0	0
COG1133	Lipid metabolism	ABC-type long-chain fatty acid transport system, fused permease and ATPase components	12	8	18	12
COG2031		Short chain fatty acids transporter	0	0	3	0
COG2067		Long-chain fatty acid transport protein	1	0	2	1
COG2867		Oligoketide cyclase/lipid transport protein	0	3	11	10
COG1953	Nucleotide transport and metabolism	Cytosine/uracil/thiamine/allantoin permeases	7	3	5	8
COG1972		Nucleoside permease	21	6	22	34
COG2233		Xanthine/uracil permeases	10	5	10	13
COG5042		Purine nucleoside permease	1	1	0	0
COG0396	Posttranslational	ABC-type transport system involved in Fe-S cluster assembly, ATPase	24	14	48	69

COG0396	modification, protein turnover, chaperones	component	24	14	48	69
COG0555		ABC-type sulfate transport system, permease component	2	0	1	1
COG0719		ABC-type transport system involved in Fe-S cluster assembly, permease component	79	34	102	185
COG0755		ABC-type transport system involved in cytochrome c biogenesis, permease component	26	6	43	73
COG2386		ABC-type transport system involved in cytochrome c biogenesis, permease component	7	1	6	10
COG4133		ABC-type transport system involved in cytochrome c biogenesis, ATPase component	3	1	5	7
COG5265		ABC-type transport system involved in Fe-S cluster assembly, permease and ATPase components	12	10	28	33
COG5238	RNA processing and modification	Ran GTPase-activating protein (RanGAP) involved in mRNA processing and transport	0	0	1	0
COG0767	Secondary metabolites biosynthesis, transport and catabolism	ABC-type transport system involved in resistance to organic solvents, permease component	6	7	22	32
COG1127		ABC-type transport system involved in resistance to organic solvents, ATPase component	3	2	19	18
COG1463		ABC-type transport system involved in resistance to organic solvents, periplasmic component	3	2	21	17
COG2854		ABC-type transport system involved in resistance to organic solvents, auxiliary component	0	2	4	4
COG3127		Predicted ABC-type transport system involved in lysophospholipase L1 biosynthesis, permease component	6	4	6	21
COG4181		Predicted ABC-type transport system involved in lysophospholipase L1 biosynthesis, ATPase component	7	1	12	15
COG4663		TRAP-type mannitol/chloroaromatic compound transport system, periplasmic component	139	40	117	126
COG4664		TRAP-type mannitol/chloroaromatic compound transport system, large permease component	37	19	105	110
COG4665		TRAP-type mannitol/chloroaromatic compound transport system, small permease component	14	4	42	35
COG3109		Activator of osmoprotectant transporter ProP	0	0	1	1
COG4191		Signal transduction histidine kinase regulating C4-dicarboxylate transport system	1	0	4	5
COG5037		Gluconate transport-inducing protein	2	0	0	1

Figure S1

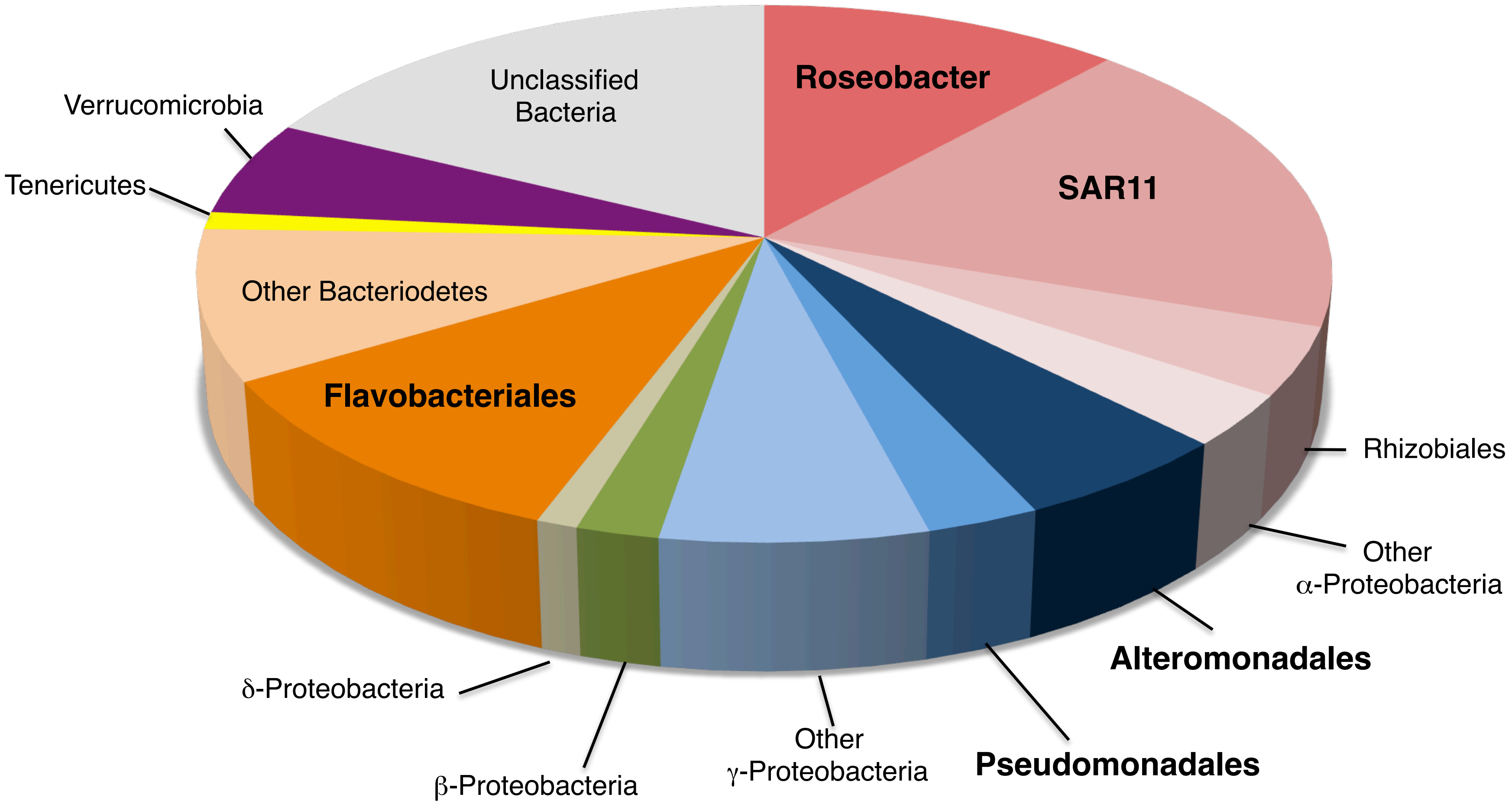

Unclassified Bacteria
Roseobacter
SAR11
Verrucomicrobia
Tenericutes
Other Bacteriodetes
Flavobacteriales
Rhizobiales
Other α-Proteobacteria
Alteromonadales
Pseudomonadales
Other γ-Proteobacteria
β-Proteobacteria
δ-Proteobacteria

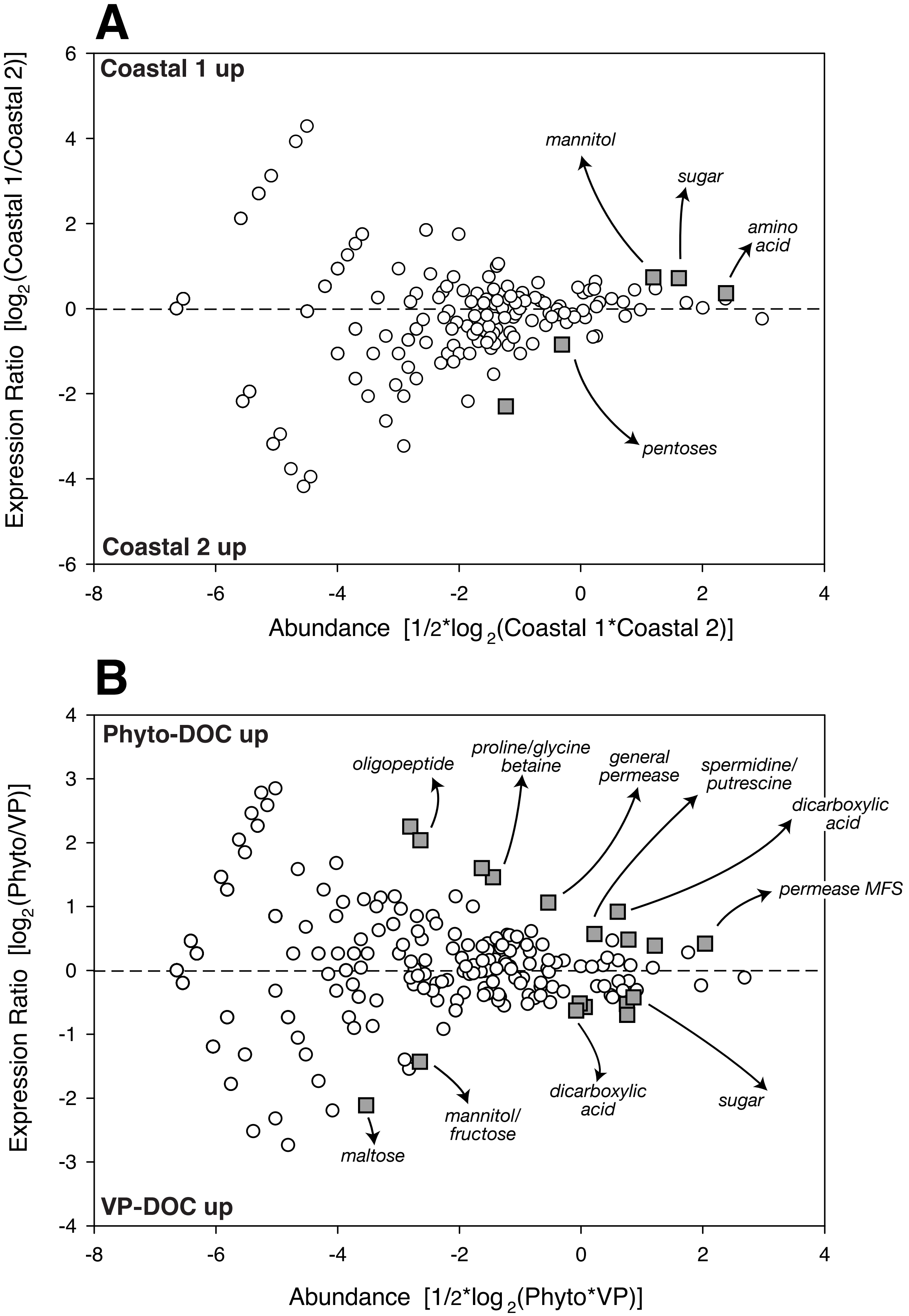
A
Coastal 1 up
Coastal 2 up
mannitol
sugar
amino acid
pentoses
Expression Ratio [log2(Coastal 1/Coastal 2)]
Abundance [1/2*log2(Coastal 1*Coastal 2)]
6
4
2
0
-2
-4
-6
-8
-6
-4
-2
0
2
4
B
Phyto-DOC up
VP-DOC up
oligopeptide
proline/glycine betaine
general permease
spermidine/ putrescine
dicarboxylic acid
permease MFS
mannitol/ fructose
maltose
dicarboxylic acid
sugar
Expression Ratio [log2(Phyto/VP)]
Abundance [1/2*log2(Phyto*VP)]
4
3
2
1
0
-1
-2
-3
-4
-8
-6
-4
-2
0
2
4

Figure S3

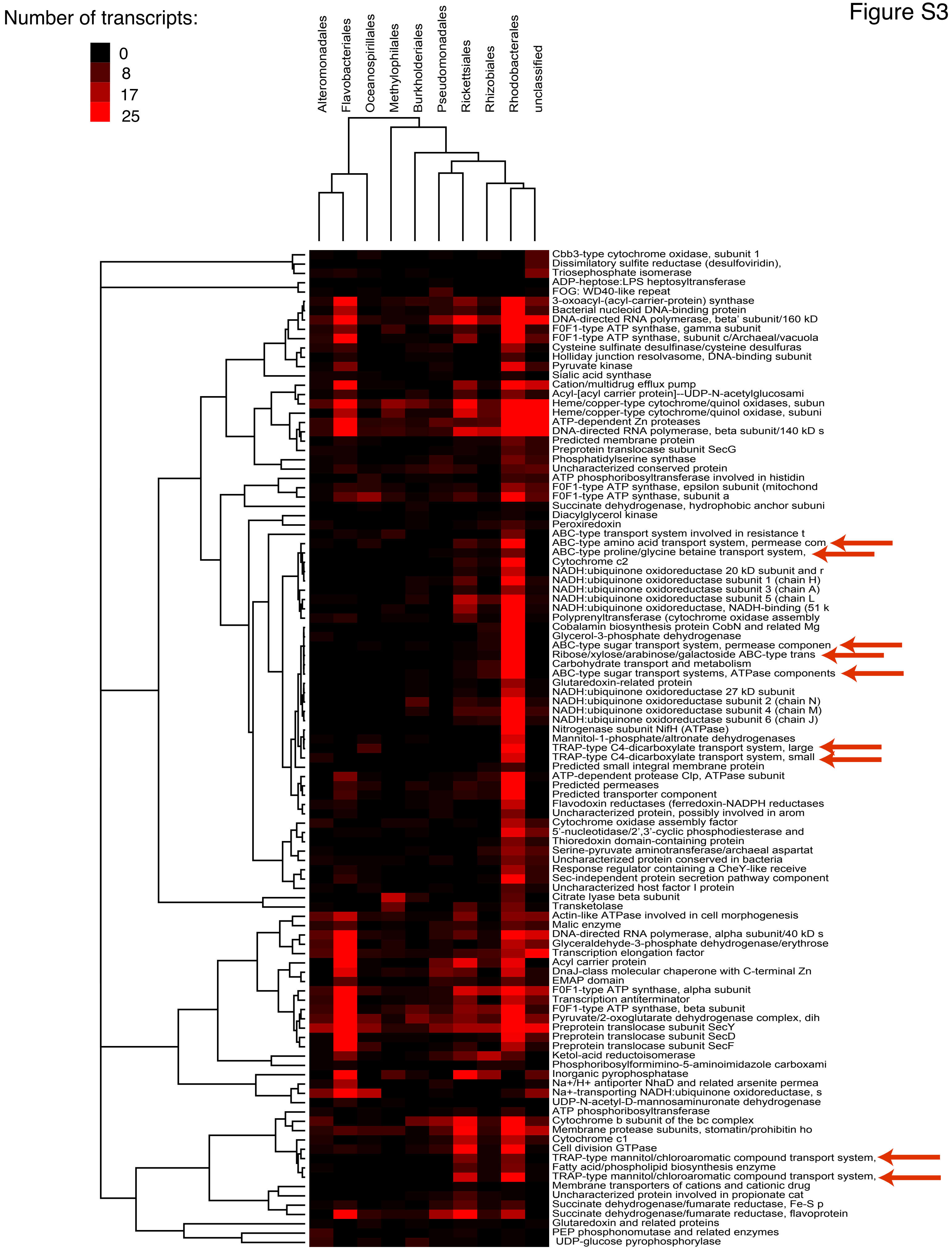

Number of transcripts:
0
8
17
25
Alteromonadales
Flavobacteriales
Oceanospirillales
Methylophilales
Burkholderiales
Pseudomonadales
Rickettsiales
Rhizobiales
Rhodobacterales
unclassified
Cbb3-type cytochrome oxidase, subunit 1
Dissimilatory sulfite reductase (desulfoviridin),
Triosephosphate isomerase
ADP-heptose:LPS heptosyltransferase
FOG: WD40-like repeat
3-oxoacyl-(acyl-carrier-protein) synthase
Bacterial nucleoid DNA-binding protein
DNA-directed RNA polymerase, beta' subunit/160 kD
F0F1-type ATP synthase, gamma subunit
F0F1-type ATP synthase, subunit c/Archaeal/vacuola
Cysteine sulfinate desulfinase/cysteine desulfuras
Holliday junction resolvasome, DNA-binding subunit
Pyruvate kinase
Sialic acid synthase
Cation/multidrug efflux pump
Acyl-[acyl carrier protein]--UDP-N-acetylglucosami
Heme/copper-type cytochrome/quinol oxidases, subun
Heme/copper-type cytochrome/quinol oxidase, subuni
ATP-dependent Zn proteases
DNA-directed RNA polymerase, beta subunit/140 kD s
Predicted membrane protein
Preprotein translocase subunit SecG
Phosphatidylserine synthase
Uncharacterized conserved protein
ATP phosphoribosyltransferase involved in histidin
F0F1-type ATP synthase, epsilon subunit (mitochond
F0F1-type ATP synthase, subunit a
Succinate dehydrogenase, hydrophobic anchor subuni
Diacylglycerol kinase
Peroxiredoxin
ABC-type transport system involved in resistance t
ABC-type amino acid transport system, permease com
ABC-type proline/glycine betaine transport system,
Cytochrome c2
NADH:ubiquinone oxidoreductase 20 kD subunit and r
NADH:ubiquinone oxidoreductase subunit 1 (chain H)
NADH:ubiquinone oxidoreductase subunit 3 (chain A)
NADH:ubiquinone oxidoreductase subunit 5 (chain L
NADH:ubiquinone oxidoreductase, NADH-binding (51 k
Polyprenyltransferase (cytochrome oxidase assembly
Cobalamin biosynthesis protein CobN and related Mg
Glycerol-3-phosphate dehydrogenase
ABC-type sugar transport system, permease componen
Ribose/xylose/arabinose/galactoside ABC-type trans
Carbohydrate transport and metabolism
ABC-type sugar transport systems, ATPase components
Glutaredoxin-related protein
NADH:ubiquinone oxidoreductase 27 kD subunit
NADH:ubiquinone oxidoreductase subunit 2 (chain N)
NADH:ubiquinone oxidoreductase subunit 4 (chain M)
NADH:ubiquinone oxidoreductase subunit 6 (chain J)
Nitrogenase subunit NifH (ATPase)
Mannitol-1-phosphate/altronate dehydrogenases
TRAP-type C4-dicarboxylate transport system, large
TRAP-type C4-dicarboxylate transport system, small
Predicted small integral membrane protein
ATP-dependent protease Clp, ATPase subunit
Predicted permeases
Predicted transporter component
Flavodoxin reductases (ferredoxin-NADPH reductases
Uncharacterized protein, possibly involved in arom
Cytochrome oxidase assembly factor
5'-nucleotidase/2',3'-cyclic phosphodiesterase and
Thioredoxin domain-containing protein
Serine-pyruvate aminotransferase/archaeal aspartat
Uncharacterized protein conserved in bacteria
Response regulator containing a CheY-like receive
Sec-independent protein secretion pathway component
Uncharacterized host factor I protein
Citrate lyase beta subunit
Transketolase
Actin-like ATPase involved in cell morphogenesis
Malic enzyme
DNA-directed RNA polymerase, alpha subunit/40 kD s
Glyceraldehyde-3-phosphate dehydrogenase/erythrose
Transcription elongation factor
Acyl carrier protein
DnaJ-class molecular chaperone with C-terminal Zn
EMAP domain
F0F1-type ATP synthase, alpha subunit
Transcription antiterminator
F0F1-type ATP synthase, beta subunit
Pyruvate/2-oxoglutarate dehydrogenase complex, dih
Preprotein translocase subunit SecY
Preprotein translocase subunit SecD
Preprotein translocase subunit SecF
Ketol-acid reductoisomerase
Phosphoribosylformimino-5-aminoimidazole carboxami
Inorganic pyrophosphatase
Na+/H+ antiporter NhaD and related arsenite permea
Na+-transporting NADH:ubiquinone oxidoreductase, s
UDP-N-acetyl-D-mannosaminuronate dehydrogenase
ATP phosphoribosyltransferase
Cytochrome b subunit of the bc complex
Membrane protease subunits, stomatin/prohibitin ho
Cytochrome c1
Cell division GTPase
TRAP-type mannitol/chloroaromatic compound transport system,
Fatty acid/phospholipid biosynthesis enzyme
TRAP-type mannitol/chloroaromatic compound transport system,
Membrane transporters of cations and cationic drug
Uncharacterized protein involved in propionate cat
Succinate dehydrogenase/fumarate reductase, Fe-S p
Succinate dehydrogenase/fumarate reductase, flavoprotein
Glutaredoxin and related proteins
PEP phosphonomutase and related enzymes
UDP-glucose pyrophosphorylase

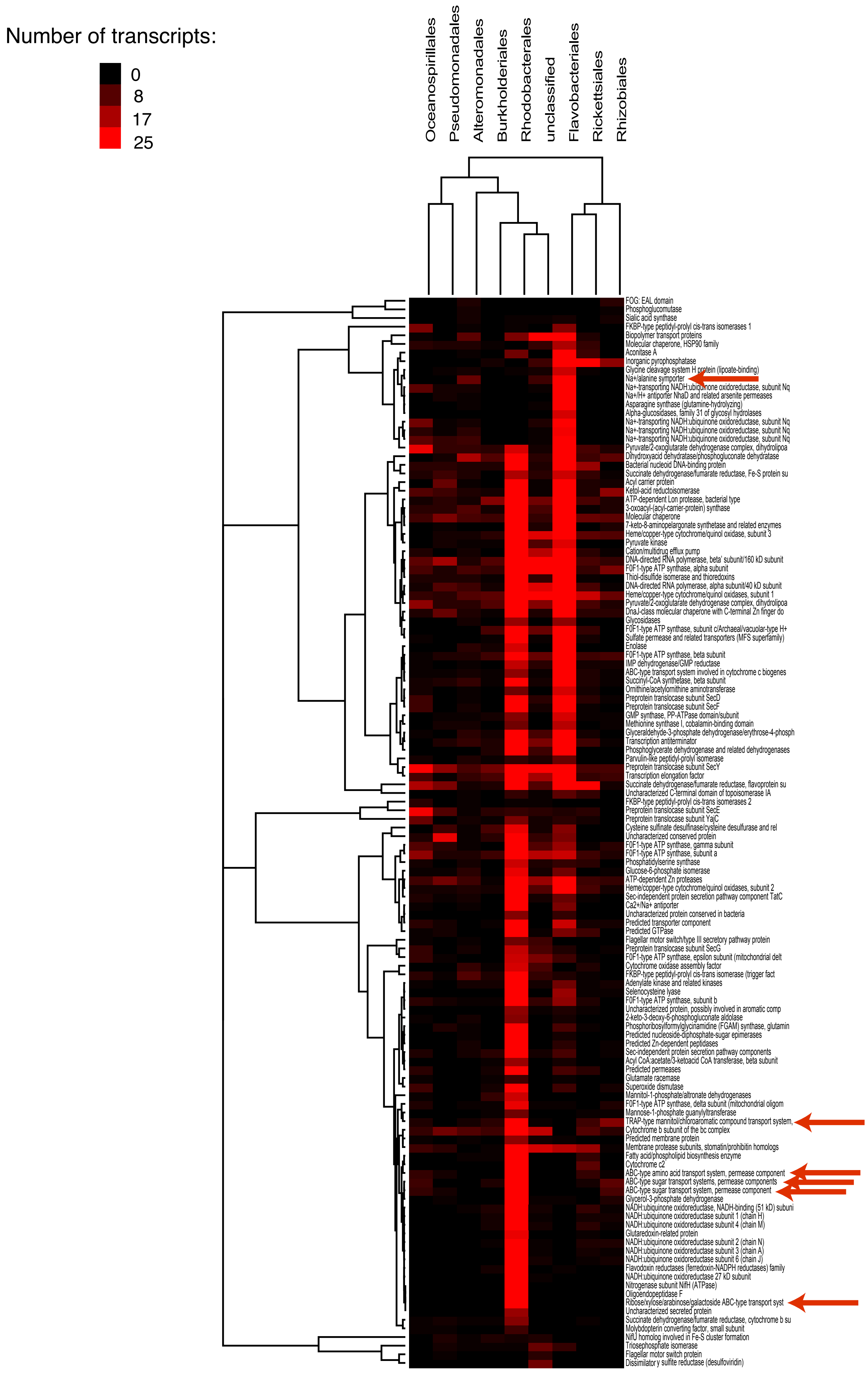
Number of transcripts:
0
8
17
25
Oceanospirillales
Pseudomonadales
Alteromonadales
Burkholderiales
Rhodobacterales
unclassified
Flavobacteriales
Rickettsiales
Rhizobiales
FOG: EAL domain
Phosphoglucomutase
Sialic acid synthase
FKBP-type peptidyl-prolyl cis-trans isomerases 1
Biopolymer transport proteins
Molecular chaperone, HSP90 family
Aconitase A
Inorganic pyrophosphatase
Glycine cleavage system H protein (lipoate-binding)
Na+/alanine symporter
Na+-transporting NADH:ubiquinone oxidoreductase, subunit Nq
Na+/H+ antiporter NhaD and related arsenite permeases
Asparagine synthase (glutamine-hydrolyzing)
Alpha-glucosidases, family 31 of glycosyl hydrolases
Na+-transporting NADH:ubiquinone oxidoreductase, subunit Nq
Na+-transporting NADH:ubiquinone oxidoreductase, subunit Nq
Na+-transporting NADH:ubiquinone oxidoreductase, subunit Nq
Pyruvate/2-oxoglutarate dehydrogenase complex, dihydrolipoa
Dihydroxyacid dehydratase/phosphogluconate dehydratase
Bacterial nucleoid DNA-binding protein
Succinate dehydrogenase/fumarate reductase, Fe-S protein su
Acyl carrier protein
Ketol-acid reductoisomerase
ATP-dependent Lon protease, bacterial type
3-oxoacyl-(acyl-carrier-protein) synthase
Molecular chaperone
7-keto-8-aminopelargonate synthetase and related enzymes
Heme/copper-type cytochrome/quinol oxidase, subunit 3
Pyruvate kinase
Cation/multidrug efflux pump
DNA-directed RNA polymerase, beta' subunit/160 kD subunit
F0F1-type ATP synthase, alpha subunit
Thiol-disulfide isomerase and thioredoxins
DNA-directed RNA polymerase, alpha subunit/40 kD subunit
Heme/copper-type cytochrome/quinol oxidases, subunit 1
Pyruvate/2-oxoglutarate dehydrogenase complex, dihydrolipoa
DnaJ-class molecular chaperone with C-terminal Zn finger do
Glycosidases
F0F1-type ATP synthase, subunit c/Archaeal/vacuolar-type H+
Sulfate permease and related transporters (MFS superfamily)
Enolase
F0F1-type ATP synthase, beta subunit
IMP dehydrogenase/GMP reductase
ABC-type transport system involved in cytochrome c biogenes
Succinyl-CoA synthetase, beta subunit
Ornithine/acetylornithine aminotransferase
Preprotein translocase subunit SecD
Preprotein translocase subunit SecF
GMP synthase, PP-ATPase domain/subunit
Methionine synthase I, cobalamin-binding domain
Glyceraldehyde-3-phosphate dehydrogenase/erythrose-4-phosph
Transcription antiterminator
Phosphoglycerate dehydrogenase and related dehydrogenases
Parvulin-like peptidyl-prolyl isomerase
Preprotein translocase subunit SecY
Transcription elongation factor
Succinate dehydrogenase/fumarate reductase, flavoprotein su
Uncharacterized C-terminal domain of topoisomerase IA
FKBP-type peptidyl-prolyl cis-trans isomerases 2
Preprotein translocase subunit SecE
Preprotein translocase subunit YajC
Cysteine sulfinate desulfinase/cysteine desulfurase and rel
Uncharacterized conserved protein
F0F1-type ATP synthase, gamma subunit
F0F1-type ATP synthase, subunit a
Phosphatidylserine synthase
Glucose-6-phosphate isomerase
ATP-dependent Zn proteases
Heme/copper-type cytochrome/quinol oxidases, subunit 2
Sec-independent protein secretion pathway component TatC
Ca2+/Na+ antiporter
Uncharacterized protein conserved in bacteria
Predicted transporter component
Predicted GTPase
Flagellar motor switch/type III secretory pathway protein
Preprotein translocase subunit SecG
F0F1-type ATP synthase, epsilon subunit (mitochondrial delt
Cytochrome oxidase assembly factor
FKBP-type peptidyl-prolyl cis-trans isomerase (trigger fact
Adenylate kinase and related kinases
Selenocysteine lyase
F0F1-type ATP synthase, subunit b
Uncharacterized protein, possibly involved in aromatic comp
2-keto-3-deoxy-6-phosphogluconate aldolase
Phosphoribosylformylglycinamidine (FGAM) synthase, glutamin
Predicted nucleoside-diphosphate-sugar epimerases
Predicted Zn-dependent peptidases
Sec-independent protein secretion pathway components
Acyl CoA:acetate/3-ketoacid CoA transferase, beta subunit
Predicted permeases
Glutamate racemase
Superoxide dismutase
Mannitol-1-phosphate/altronate dehydrogenases
F0F1-type ATP synthase, delta subunit (mitochondrial oligom
Mannose-1-phosphate guanylyltransferase
TRAP-type mannitol/chloroaromatic compound transport system,
Cytochrome b subunit of the bc complex
Predicted membrane protein
Membrane protease subunits, stomatin/prohibitin homologs
Fatty acid/phospholipid biosynthesis enzyme
Cytochrome c2
ABC-type amino acid transport system, permease component
ABC-type sugar transport systems, permease components
ABC-type sugar transport system, permease component
Glycerol-3-phosphate dehydrogenase
NADH:ubiquinone oxidoreductase, NADH-binding (51 kD) subuni
NADH:ubiquinone oxidoreductase subunit 1 (chain H)
NADH:ubiquinone oxidoreductase subunit 4 (chain M)
Glutaredoxin-related protein
NADH:ubiquinone oxidoreductase subunit 2 (chain N)
NADH:ubiquinone oxidoreductase subunit 3 (chain A)
NADH:ubiquinone oxidoreductase subunit 6 (chain J)
Flavodoxin reductases (ferredoxin-NADPH reductases) family
NADH:ubiquinone oxidoreductase 27 kD subunit
Nitrogenase subunit NifH (ATPase)
Oligoendopeptidase F
Ribose/xylose/arabinose/galactoside ABC-type transport syst
Uncharacterized secreted protein
Succinate dehydrogenase/fumarate reductase, cytochrome b su
Molybdopterin converting factor, small subunit
NifU homolog involved in Fe-S cluster formation
Triosephosphate isomerase
Flagellar motor switch protein
Dissimilatory sulfite reductase (desulfoviridin)

Figure S4
